# Thiol-disulfide Oxidoreductases TRX1 and TMX3 Decrease Neuronal Atrophy in a Lentiviral Mouse Model of Huntington’s Disease

**DOI:** 10.1371/currents.hd.b966ec2eca8e2d89d2bb4d020be4351e

**Published:** 2015-11-06

**Authors:** Jonathan Fox, Zhen Lu, Lorraine Barrows

**Affiliations:** Neuroscience Graduate Program, Department of Veterinary Sciences, University of Wyoming, Laramie, Wyoming, USA; Neuroscience Graduate Program, Department of Veterinary Sciences, University of Wyoming, Laramie, Wyoming, USA

## Abstract

Huntington’s disease (HD) is caused by a trinucleotide CAG repeat in the huntingtin gene (HTT) that results in expression of a polyglutamine-expanded mutant huntingtin protein (mHTT). N-terminal fragments of mHTT accumulate in brain neurons and glia as soluble monomeric and oligomeric species as well as insoluble protein aggregates and drive the disease process. Decreasing mHTT levels in brain provides protection and reversal of disease signs in HD mice making mHTT a prime target for disease modification. There is evidence for aberrant thiol oxidation within mHTT and other proteins in HD models. Based on this, we hypothesized that a specific thiol-disulfide oxidoreductase exists that decreases mHTT levels in cells and provides protection in HD mice. We undertook an in-vitro genetic screen of key thiol-disulfide oxidoreductases then completed secondary screens to identify those with mHTT decreasing properties. Our in-vitro experiments identified thioredoxin 1 and thioredoxin-related transmembrane protein 3 as proteins that decrease soluble mHTT levels in cultured cells. Using a lentiviral mouse model of HD we tested the effect of these proteins in striatum. Both proteins decreased mHTT-induced striatal neuronal atrophy. Findings provide evidence for a role of dysregulated protein-thiol homeostasis in the pathogenesis of HD.

## Introduction

Huntington’s disease (HD) is a progressive autosomal dominant neurodegenerative disorder caused by a trinucleotide CAG repeat expansion in exon-1 of the huntingtin gene (*HTT*)^1^. Disease onset is typically in early to mid-adult life with a range from childhood to advanced age. Clinical signs of HD include involuntary movements, psychiatric problems and cognitive decline. Motor signs of HD result largely from dysfunction and loss of neurons in striatum and cerebral cortex^2^^,^ 3. Treatments that delay onset or progression of human HD have yet to be developed.

Trinucleotide-repeat expansion within the *HTT* gene results in expression of a polyglutamine-expanded mutant huntingtin protein (mHTT). Full-length soluble mHTT undergoes enzymatic cleavage to generate soluble N-terminal mHTT polyglutamine containing fragments^4^^,^ 5. Mutant huntingtin N-terminal fragments exist as monomers, soluble oligomers and larger insoluble aggregates^6^. Soluble N-terminal mHTT fragments are thought to be the main drivers of disease progression^7^ and mouse models of HD that express these fragments have a rapidly progressive phenotype^8^. N-terminal mHTT results in HD through accumulation in cells and aberrant interactions with numerous proteins^9^^,^ 10 and possibly direct production of reactive oxygen species^11^. Genetic therapeutic approaches that decrease mHTT levels offer the possibility of inhibiting downstream disease pathways, reversing the disease process, and are a promising approach for future treatment of human HD^12^^,^ 13 ^,^ 14 ^,^ 15 .

We have previously reported that the toxic N-terminal 171 fragment of mHTT is prone to thiol oxidation resulting in the formation of soluble oligomeric mHTT; furthermore, these oligomers are degraded more slowly than monomeric mHTT^6^. Therefore, thiol oxidation within mHTT may contribute to cellular accumulation and toxicity. There is accumulating evidence for the dysregulation of thiol homeostasis in HD. For example, S-nitrosylation of dynamin-related protein 1, a GTPase that mediates mitochondria fission, has been shown to promote degeneration in HD models^16^. Another study in cultured HD cells showed increased thiol oxidation of peroxiredoxin 1, a protein involved in removal of hydrogen and lipid peroxides^17^. Furthermore, there are increased levels of copper and iron in mouse HD brain, which in unbound form, can promote thiol oxidation^18^ ^19^^,^ 20.

Thiol-disulfide oxidoreductase enzymes mediate protein repair and folding processes. They share a common C-X-X-C catalytic sequence within a thioredoxin-type domain that is required for enzymatic activity and they reduce disulfides by forming a catalytic-site disulfide which is then reduced by an external electron donor^21^. These enzymes differ in cell location, protein substrates and mechanism of reactivation. Thioredoxins are small thiol-disulfide oxidoreductases involved in repair of oxidatively damaged thiols and redox regulation of cell-signaling pathways via thiol switches^22^^,^ 23. Glutaredoxins have thiol reductase and deglutathioylation activity; they are required for normal mitochondrial function and protection against neurodegenerative processes^24^^,^ 25 ^,^ 26. Protein-disulfide isomerases are another group of enzymes with a thioredoxin domain; they are located in the endoplasmic reticulum (ER) and other compartments where they regulate a large number of processes via disulfide exchange reactions^27^. Collectively, these oxidoreductases regulate protein folding in the cell secretory pathway, modulation of activity within cell signaling pathways, and repair of oxidatively-damaged nuclear, cytoplasmic and mitochondrial proteins (reviewed^24^^,^ 28).

Mutant huntingtin undergoes a number of post-translational modifications. Phosphorylation status of serine 13 and 16 within the N-terminus of huntingtin protein is a critical determinant of HD^29^. Furthermore, acetylation of mHTT lysine 444, down-stream of the glutamine expansion, promotes mHTT clearance by increasing trafficking to the autophagosome^30^. These post-translational modifications suggest potential therapeutic approaches for modifying HD proximally at the level of mHTT. We have reasoned that there may be a thiol-disulfide oxidoreductase that has protective effects by decreasing levels of N171 mHTT, possibly by direct activity on mHTT protein. We therefore undertook a study to seek a thiol-disulfide oxidoreductase with mHTT lowering effects in HD cells that we could test in HD mice. We found that thioredoxin 1 (TRX1) and thioredoxin-related transmembrane protein 3 (TMX3) both decreased levels of mHTT in cells but did not find evidence for a direct interaction with mHTT. Using a lentiviral mouse model expressing N171 mHTT^31^, we found that TRX1 and TMX3 decreased striatal neuronal atrophy. Findings support a modulatory role of TRX1 and TMX3 in these HD model systems.

## Materials and Methods

**Materials:** Mouse anti-huntingtin (HTT) (MAB5374) was from Chemicon, and mouse monoclonal anti-β-actin antibody (AC40) from Sigma. Unless otherwise stated all chemicals were from Sigma.

**Primary screen for mutant HTT protein lowering thiol-disulfide oxidoreductases:** COS-1 cells were grown in DMEM supplemented with 10% fetal bovine serum (FBS), 1% L-glutamine and 1% penicillin and streptomycin at 37⁰C and 5% CO2. For experiments cells were grown in 12-well plates and transfected with plasmid(s) using lipofectamine 2000 (Life Technologies) and reduced serum medium (OPTI-MEM®-1; Life Technologies) at 70-80% confluency using standard procedures. N171-40Q was in pcDNA1 vector and those encoding thiol-disulfide oxidoreductases were in pQE-TRiSystem vector (Qiagen) and expressed with a polyhistidine tag. Gene accession numbers are: NM_001118890.1, NM_016066.4, NM_006541.4, NM_016417.2, NM_001080476.2, NM_001080516.1, NM_001164478.1, NM_003329.1, NM_012473.3, NM_019022.3, NM_021156.3, NM_005742.3, NM_015051.2, NM_004261.3 and NM_080430.2. For plasmids encoding N171-40Q and the thiol-disulfide oxidoreductases 830 ng of each plasmid DNA was used. We co-transfected with plasmids encoding N171-40Q and GFP as a control. Cells were lysed 48 hours after transfection and levels of soluble N171-40Q and actin were measured by Western blot analysis (see below). Actin normalized values were then determined.

**Secondary screen for mutant HTT protein lowering thiol-disulfide oxidoreductase:** To provide a robust control for each candidate proteins enzymatic activity, we expressed as a control the same protein but with mutation of active-site residues that blocks activity. Thiol-disulfide oxidoreducases have a C-X-X-C active-site and share a common catalytic mechanism. PDIA6 has two C-X-X-C motifs while the others have one. In one study mutation of the N-terminal cysteine within this motif completely blocked enzymatic activity while mutation of the lower cysteine inhibited activity by 90% [32]. Therefore, we replaced the N-terminal active-site cysteine with serine to generate enzymatically inactive control proteins. We generated constructs expressing enzymatically inactive TRX1, TMX3, GLRX1, PDIA6 and FLJ44606 using QuikChange® site-directed mutagenesis kit (Stratagene) for use as a specific control for each candidate test protein. Constructs were verified by DNA sequencing. COS1 cells were transfected with plasmids encoding active or inactive thiol-disulfide oxidoreductase, and then N171-40Q levels determined by Western blot analysis.

**Western blot analysis:** For cell culture experiments, cells were washed in cold PBS then lysed directly in lysis buffer [20 mM TRIS (pH 7.4), 1 mM EDTA, 0.15 M NaCl, 0.1% Triton-x100 and protease inhibitor cocktail]. Thirty µg protein samples were resolved by reducing SDS-PAGE. Proteins were transferred to PVDF, blocked with 5% non-fat milk in Tris-buffered saline containing 0.1% Tween (TBS-T) at room temperature for 1 hour then incubated with anti-HTT (MAB5492 – 1:2000 dilution) and anti-actin (AC40 – 1:2000) overnight at 4⁰C. After the primary incubation, membranes were washed 4 times for 10 minutes in TBS-T and incubated with goat polyclonal to mouse IgG HRP (Abcam, 1:2000 dilution) at room temperature for an hour. Then membranes were washed again and placed in Western Blotting Luminol Reagent (Santa Cruz Biotechnology, Inc.) before imaging with a CP1000 Film Processor (AGFA). Image J software (NIH) was used to quantify band density. Total HTT protein levels were determined by the ratios of the values for HTT and β-actin. For mouse studies, mice were anesthetized and perfused with cold-heparinized 0.9% (w/v) saline. Brains were sectioned frontally at a 1 mm interval then two sections were taken at the level of the injection site, striatum dissected and then frozen on dry ice before storing at -80°C. Brains were homogenized in lysis buffer which were then incubated on ice for 5 minutes then centrifuged for 10 minutes at 16 000 x g and 4⁰C. Protein concentrations were determined then Western blot analysis performed as described above.

**N171 HTT protein TRX1 interaction experiment:** We used a previously described approach to test whether N171 mHTT is a direct substrate of TRX1^33^^,^ 34. In brief, a variant of TRX1 with a mutation of the lower cysteine residue within the CXXC active-site motif is used to trap the catalytic disulfide-linked heterodimer with the substrate protein. Heterodimers can be detected by non-reducing Western blot analysis. DNA encoding N171-40Q, TRX1 and mutant TRX1 (mTRX1) was sub-cloned into the bacterial expression vector pGEX-6P-1 (GE Healthcare). Proteins were expressed in bacteria, purified using a GST column, then GST removed by cleavage with PreScission Protease (GE Healthcare) as previously described^6^ . The purified protein was buffer exchanged into 50 mM Tris (pH 7.0) and 150 mM NaCl. N171-40Q was incubated with TXN or mutant TXN at room temperature for one hour; 50 mM N-ethylmaleimide was then added to block free thiols and the samples were then resolved by reducing or non-reducing SDS-PAGE and proteins detected by western blot analysis.

**Mouse husbandry:** All procedures and euthanasia methods were approved by the University of Wyoming Institutional Animal Care and Use Committee and were also in accordance with NIH guidelines. We used female B6/C3H F1 mice purchased from the Jackson Laboratory. Mice were maintained under standard conditions of housing and lighting. They were fed a standard cereal-based rodent chow and had ad-libitum access to acidified water (pH 3-4).

**Mouse study experimental design:** C57BL/6 x C3H F1 female mice were purchased at 5-6 weeks of age. Pre-treatment wheel analysis was at 7 weeks of age. Surgeries were at ~8 weeks of age and mice were sacrificed 6-weeks later based on the studies of de Almeida et al^31^.

**Lentiviral synthesis:** We utilized a four-plasmid system for generation of lentivirus expressing N171-18Q and N171-82Q (kindly provided by Dr. Deglon). We subcloned DNA encoding functionally active and inactive versions of TRX1 and TMX3 from pQE-Tri plasmids into the SIN-pGK which uses the phosphoglycerate kinase promoter to drive gene expression. For each virus, the four plasmids were transfected in the molar ratio of 1:1:1:3 for pMDG, CMVΔ8.92, pRSV-Rev and SIN-pGK, respectively. For each T-150 sized flask we used 3.3, 7.2, 2.2 and 7.2 µg plasmid. 293T cells were grown to ~60% confluency in 10% FBS-DMEM and 2 mM glutamine. They were then transfected using jetPrime transfection reagent (Polyplus 114-07) according to the manufacturer’s instructions. For each T-150 flask we used 1 ml of jetPrime buffer, 20 µg combined plasmids, 50 jetPrime reagent and 36 mls of cell culture medium. Cells were transfected for four hours, washed twice in PBS then the medium replaced. Cells were incubated for a further 72 hours prior to virus purification. Medium was harvested and placed into sterile tubes on wet ice then filtered using a 0.22 µm filter. The filtered supernatant was centrifuged in a SW28 swinging bucket rotor on a Beckman L8-80 centrifuge at 141k x g for 2 hours at 4 degrees C. Supernatant was decanted then tubes were inverted for 4 minutes. The pellet was re-suspended in 300-500 µl of sterile PBS then transferred to a siliconized tube. Samples were then centrifuged at 19000 x g for 60 min at 4 degrees C to concentrate then the pellet resuspended in 50-150 µl PBS by gentle pipetting. The samples were then mixed gently overnight using a tilted horizontal shaker at 4 degrees C. Lentiviruses were quantified in duplicate using a p24 ELISA (Zeptometrix) according to the manufacturer’s instructions. Lentiviral samples were diluted to 4.0 ng p24 / µl in PBS, stored at 4 degrees C in siliconized tubes and used within 2 weeks of preparation.

**Stereotaxic surgery:** Mice were anesthetized with 1.75 mg ketamine and 0.25 mg xylazine per 20 grams body weight. After mounting in a stereotaxic frame they were injected at the following coordinates: AP =+0.4; DV= -3.9, then pull up to -3.7 before injection; ML= (all to right), +2.0 if body weight ≥22.5 g, 1.95 if body weight = 20.5-22.4 g, 1.85 if body weight = 19.0-20.4 g, and 1.75 if body weight = 17.5-18.9 g. The needle size used was ½ inch, 31 gauge, and with a 30 degree bevel. Virus was delivered with a peristaltic pump at a rate of 200 nl per minute (2.5 µl over 12.5 minutes). The needle was then left in place an additional 10 minutes before slow removal. Each mouse was injected with 10 ng of p24 equivalents of virus. For testing paired viral injections, different combinations of virus were injected; we used 5 ng of p24 equivalents of each virus which were pre-mixed prior to injection.

**Wheel activity analysis:** Spontaneous wheel activity was measured before and after surgery. Mice were placed individually in cages containing running wheels for 4 days, running times in both light and dark cycles (12 hours each) were recorded. The first day was used to familiarize mice with the instrument. Data from days 2-4 were used for analysis.

**Immunofluorescence staining and brain stereology:** Mice were sacrificed by an intra-peritoneal overdose of phenytoin and pentobarbital solution. This was followed by a 2 minute perfusion in heparinized 0.9% saline immediately followed by perfusion of 200 mls of freshly prepared 4% paraformaldehyde (in 0.1 M phosphate buffer, pH 7.5). Brains were removed 1-4 hours later and immersion fixed for a further 24 hours before being transferred to cryo-preservant (10% glycerol in 0.1 M phosphate buffer, pH 7.5). Brains were sectioned frontally at 40 μm and stored in 0.1 M phosphate buffer (pH 7.5) containing 0.05% azide at 4°C. Striatal sections at the level of the anterior commissure were incubated with primary anti-huntingtin antibody EM48 (Chemicon) at 1:1000 dilution in PBS-0.1% tween-10% goat serum for 3 days at 4°C, then followed by a 24 hour incubation with Alexa-fluor 488 labeled secondary antibody at 1:500 dilution (Life Technologies). Sections were washed in PBS three times for 10 minutes and stained with fluorescent Nissl (Life Technologies) for 1 hour at room temperature (1:100 dilution in PBS), followed by washing in PBS twice for 15 minutes and mounting on slides using Fluoromount G (Southern Biotech). Slides were air-dried overnight in the dark and then stored at 4°C. Nissl stain was used to detect neuronal cell bodies at 640/660 nm. Images were captured using a Zeiss 710 confocal microscope. We stained several striatal sections per mouse to identify sections containing expressed protein. Within each region of positive mHTT staining 2-3 image stacks were obtained using a 60x objective (z-stack interval = 0.46 µm). Within these images we quantified all striatal neurons regardless of whether there was mHTT staining as suppression of mHTT levels by TRX1 or TMX3 could result in loss of detectable staining. Striatal neuronal volumes were estimated using the confocal module in Stereoinvestigator software (MicroBrightField, Williston, VT) and the five-ray nucleator method.

**Quantitative PCR for TMX3 and TXN1 in N171-82Q HD mice:** We used essentially the same method as previously described^35^.

**Statistical analyses:** Data was analyzed with SAS software version 9. Student’s t-test were used for the analysis of the secondary screens. One-way ANOVA was used for the analysis striatal neuronal volumes. Repeated-measures ANOVA was used to analyze spontaneous wheel-running data. P-values less than 0.05 were considered significant.

## Results

**Identification of TRX1 and TMX3 as candidate mHTT decreasing proteins in HD**

We studied a broad range of human thiol-disulfide oxidoreductase genes. This group includes all the well-studied thiol-disulfide oxidoreductases to include the glutaredoxin, thioredoxin and thioredoxin reductase gene families. There are a growing number of poorly characterized proteins considered to have a thioredoxin-like domain and probable thiol-disulfide oxidoreductase activity. We studied several of these proteins to include the thioredoxin-related transmembrane proteins and thioredoxin-like proteins. COS1 cells were co-transfected with plasmids encoding the N171-40Q fragment of mHTT protein together with a plasmid encoding the test thiol-disulfide oxidoreductase. Co-transfection with plasmids encoding N171-40Q mHTT and GFP were used as controls. Cells were lysed 24 hours post-transfection and analyzed for soluble mHTT levels. For each gene studied, N171-40Q expression was normalized to actin then the result normalized to the N171-40Q/GFP control (100%). As shown in Fig. 1A, we found markedly different effects of test thiol-disulfide oxidoreductases on soluble N171-40Q mHTT levels. However, we subsequently found that co-transfection with GFP significantly suppressed N171-40Q protein levels (not shown). Therefore, rather than comparing test genes with the N171-40Q/GFP baseline, we qualitatively selected genes for more detailed testing based on them resulting in low or high N171-40Q expression levels, compared to other candidates. Thioredoxin 1 (TRX1) and thioredoxin-related transmembrane protein 3 (TMX3) were chosen as candidates that may decrease N171-40Q levels. Glutaredoxin 1 (GLX1), protein disulfide isomerase family A, member 6 (PDIA6) and FLJ44606 were chosen as candidates that may increase mHTT. Sel-M and Sel-15 are selenoproteins that were also mHTT decreasing candidates; they were not included in subsequent investigations. To provide a more rigorous assessment of the selected candidates we developed a secondary screening assay to further validate results. The candidate proteins share a common thioredoxin-domain structure with the same catalytic mechanism that involves a C-X-X-C motif. Further, mutation of the first cysteine residue of this site blocks enzymatic activity^32^. To specifically assess the effect of thiol-disulfide oxidoreductase enzymatic activity on mHTT levels in our secondary screen we compared the effect of active protein with protein in which enzymatic activity had been blocked by replacement of the critical cysteine with serine (see methods). As shown in Fig. 1B-D GLX1 (1B) increased mHTT (p<0.05); TRX1 (1C) decreased mHTT (p<0.05); and TMX3 (1D) decreased mHTT (p<0.01) consistent with the findings from the primary screen (Fig. 1A). However, active LJ44606 (1E) and PDIA6 (1F) had no effect on mHTT levels (p>0.05). GLX1 increased N171-40Q protein levels (1B) suggesting that GLX1 inhibitors may decrease mHTT. However, while GLX1 knockout is not lethal in mice^36^ the protein is required for normal mitochondrial function^26^. As GLX1 inhibitors would be predicted to be toxic, we excluded it from further investigation.

To further elucidate mechanisms for decreasing mHTT levels in cells we used two approaches. First, we tested to determine if TRX1 and TMX3 can decrease levels of a N171-40Q variant that lacks thiol-containing cysteine residues and does not form reduction-sensitive oligomers. We used a plasmid encoding N171-40Q-4CA in which all four cysteine residues are mutated to block formation of thiol-dependent oligomers^6^. In co-transfection experiments there was no effect of TRX1 and TMX3 on decreasing N171-40Q-4CA as compared to mutant TRX1 and mutant TMX3 controls (Fig. 2A-B). Infact, functional TMX3 increased N171-40Q-4CA compared to mutant TMX3 control (p<0.05) (Fig. 2B). The reason for this is unclear. However, the lack of decrease of N171-40Q-4CA by active TXN1 and TMX3 suggests that the decreasing effects of these thiol-disulfide oxidoreductase on N171-40Q levels depends on the presence of N171 HTT thiols. TRX1 is a cytosolic and nuclear protein and could therefore have direct contact with HTT^37^^,^ 38. We therefore addressed if N171-40Q mHTT is a direct substrate of TRX1. We used a previously described method that can trap TRX1 with its substrate in a catalytic intermediate state [34] . As shown in Fig. 3 we found no evidence that N171-40Q mHTT is a direct substrate of TRX1 under cell-free conditions. Similar experiments using transfected COS cells also failed to find evidence of a direct interaction between N171 HTT and TRX1 (not shown). TMX3 is a transmembrane protein with its active site within the endoplasmic reticulum^39^. As there is no evidence that HTT is an endoplasmic reticulum luminal protein we did not test for a direct TMX3 HTT interaction. However, as both TRX1 and TMX3 could be protective by effects on non-HTT targets we tested them in mouse HD.

**Validation of a lentiviral mouse model of HD**

Lentiviral vectors have been used to model HD in rats and macaques^40^. We used a four plasmid lentiviral system previously described by de Almeida et al in rats^31^ to model HD in wild-type mice. This system drives expression of the N171 HTT fragment under the control of the phosphoglycerate kinase promoter providing neuronal expression at physiologically relevant protein levels. Mice were injected with virus at ~8 weeks and sacrificed as ~14 weeks of age. Lentiviral-mediated expression of N171-18Q and 82Q HTT resulted in detectable expression by immunofluorescence staining of brain sections (Fig. 4A). However, neuronal expression was only found immediately around the needle tract. Western blot analysis failed to detect N171-18Q but detected N171-82Q (Fig. 4B). Therefore, both wild-type and mutant N171 protein were expressed in brain; higher expression of mHTT may be related to its accumulation as part of the disease process^41^. We chose to use striatal neuronal cell body volume as our main outcome as cell atrophy is a consistent manifestation of mHTT expression in neurons^42^. In this preliminary study, while differences were not statistically significant (p=0.2202) striatal neuronal cell body volume means were lower in the N171-82Q (n=11) versus N171-18Q (n=5) group (520 ± 53 and 639 ± 78 µm3, respectively).

**TRX1 and TMX3 decrease neuronal atrophy in mouse HD**

We sub-cloned cDNA-encoding TMX3 and TRX1 into the same lentiviral expression system used for N171 expression^31^ then generated enzymatically inactive variants by point mutagenesis for use as gene-specific controls for enzymatic activity of the test protein. We removed the histidine tags during the sub-cloning process. While we did not attempt to show protein expression in brain, we did demonstrate in-vivo transcript expression of TMX3 and TRX1 in liver 6 weeks after intra-venous injection of neonatal mice (not shown). We then undertook experiments in which we compared the effects of N171-18Q, N171-82Q, and N171-82Q with TMX3 or TRX1 following mouse striatal injection. To control for the total level of lentiviral delivery to striatum we co-injected virus encoding mutant (inactive) TMX3 or TRX1 in the N171-18Q and N171-82Q treatment groups. Striatal injections were at ~8 weeks and mice were sacrificed at ~14 weeks of age. We completed confocal stereology to quantify striatal neuronal cell body volume. As our candidate treatments were chosen based on their ability to decrease mHTT it is possible that mHTT would not be detected in an infected cell by immunofluorescence and that the transduced cell would not be quantified. Therefore, we changed our methodologic approach. We stained brain sections for mHTT then captured confocal images in regions where there was some neuronal mHTT staining. We then quantified cell body volume for all neurons within an image stack. As shown in Fig. 5 (TMX3 experiment) and Fig. 6 (TRX1 experiment) we found that striatal neuronal cell body volumes were significantly decreased in N171-82Q versus N171-18Q expressing mice (p-values: 0.0350 and 0.0035, respectively). Functionally active TMX3 and TRX1 both decreased this effect of mHTT on striatal neuronal atrophy (p-values: 0.0387 and 0.0046, respectively). Due to the presence of detectable mHTT staining only around the needle tract quantification of soluble mHTT levels by Western blot analysis following brain dissection would not have been reliable therefore we did not attempt this. Finally, we studied TRX1 and TMX3 transcript levels by qPCR in 14-week old N171-82Q transgenic HD mice (equivalent to early-advanced disease^35^); these mice express the N171-82Q HTT fragment under the control of the prion promoter^8^ (Fig. 7). There was no evidence of decreased expression of both genes in striatum and cerebral cortex; in fact, in striatal TRX1 transcript expression was significantly increased in HD mice (p<0.01) (Fig. 7A). We additionally completed searches of publically available HD micro-array data sets using GeoProfiles. In the R6/1 mouse model of HD there was an increase in cerebral TXN1 transcript from 22-27 weeks of age with 1 of 2 probes; TMX3 transcript changes were not found^43^. Micro-array analysis in 12 and 24 month old full-length mHTT expressing YAC128 HD and wild-type litter-mate striata did not reveal expression differences for TXN1 or TMX3^44^.

## Discussion

Abnormal redox homeostasis and oxidative stress are consistent features of human HD and cell-based and animal models^45^^,^ 46. Identification of appropriate targets for modulation of redox homeostasis could provide novel therapeutic approaches for treating HD. Protein thiols are an important site of post-translational modification involved in the regulation of redox responsive cell signaling processes^24^. Oxidative stress can result in increased protein thiol oxidation and disruption of these homeostatic processes, potentially contributing to cell dysfunction and degeneration. Transgenic mice expressing the N171 mHTT protein fragment develop a phenotype similar to human HD including striatal atrophy^47^. We have shown that the N171 fragment of HTT can form thiol-dependent oligomers which are degraded more slowly than a N171 protein variant that lacks thiols and is unable to oligomerize^6^. Numerous proteins with thiol-disulfide oxidoreductase activity exist that facilitate the reduction of oxidized protein thiols in cells^24^. Here we sought to identify if there are thiol-disulfide oxidoreductase enzymes that can decrease mHTT levels in cells and provide protection against neuronal atrophy in HD mice. We tested a representative set of thiol-disulfide oxidoreductases for mHTT decreasing effects. We used primary and secondary cell-based screens to identify candidate genes for testing in HD mice (Fig. 1). Based on our previous findings^6^ enzymatic conversion of mHTT oligomer to monomer by a thiol-disulfide oxidoreductase is expected to result in increased monomer degradation and potentially no change in monomer to oligomer ratio; we therefore quantified total soluble mHTT levels by reducing SDS-PAGE in our cell-based studies. To enable timely progression to in-vivo testing we utilized a lentiviral system to drive expression of N171 mHTT and test our candidate genes in mouse brain (Figs. 5-6).

Consistent with reports of lentivirus-induced HD in rats showing no behavioral changes^31^, effects of striatal expression of N171-82Q mHTT in mice on spontaneous wheel running activity were not found (Figs. 5-6). N171 HTT expression was found mainly around the needle tract suggesting transduction of a small percentage of the overall striatal volume potentially explaining the lack of a behavioral phenotype. However, by characterizing somal volume of neurons we were able to obtain a measure of the effect of mHTT and test proteins TRX1 and TMX3. Neuronal atrophy is a consistent morphologic feature of HD and is frequently used as a marker of therapeutic effect 42. Based on this outcome, we provide evidence that both TRX1 and TMX3 have protective effects in the lentiviral mouse HD system tested (Figs. 5-6).

TRX1 is a well-characterized cytoplasmic and nuclear thiol-disulfide oxidoreductase that has previously been demonstrated to have protective effects in models of acute and chronic neurodegeneration. TRX1 transgenic mice demonstrate increased resistance to neuronal degeneration induced by transient focal ischemia^48^. TRX1 interacting protein (TXNIP) is an endogenous inhibitor of TRX1 and is expressed in brain^49^. Furthermore, TXNIP inhibitors provide protection in a rodent model of thromboembolic stroke^50^. TRX1 has also been shown to promote neurogenesis and cognitive recovery following cerebral ischemia in mice^51^. DJ-1 is an anti-oxidant protein; mutations in DJ-1 cause autosomal recessive early-onset Parkinson’s disease^52^. In one study, it was shown that DJ-1 mediates its neuroprotective effects by stimulating Nrf2-mediated upregulation of TRX1^53^. The protective effect of 17β-estradiol in the tumor necrosis factor model of optic neuropathy is also mediated by TRX1^54^. Protective effects of TRX1 in disparate models of neuronal degeneration are consistent with its key role in redox regulation of signaling pathways and repair of oxidatively-modified thiols within diverse proteins. The current findings demonstrate that protective effects of TRX1 also extend to a model of mouse HD.

To determine if the effect of TRX1 on decreasing N171 mHTT (Fig. 1) is the result of a direct effect of TRX1 on N171-40Q we used a previously reported approach that utilizes a TRX1 variant to trap the intermediate catalytic state of TRX1 di-sulfide linked to its substrate protein as a heterodimer^34^. We used purified N171-40Q HTT and TRX1 or mutant TRX1 in a cell-free assay to maximize the chances of finding an interaction. Despite this, we found no evidence that N171-40Q HTT is a direct substrate of TRX1 (Fig. 3). As this result could be because the proteins expressed in bacteria failed to fold properly, we undertook similar experiments in transfected COS cells but also failed to find evidence for a disulfide-linked heterodimer species (not shown). Therefore, while we cannot fully exclude the possibility, we have no data to indicate that N171 HTT disulfides^55^ may be a direct substrate of TXN1.

TRX1 has many substrate proteins^23^^,^ 56. Therefore, the neuronal atrophy decreasing effect of TRX1 that we observed (Fig. 6) may be the result of effects on non-HTT targets. Peroxiredoxins are a family of redox proteins that regulate hydrogen and lipid peroxide levels by oxidation of catalytic cysteine thiols then subsequent reductive re-activation. Thioredoxins activate oxidized peroxiredoxins^57^. Importantly, it has been shown in a rat cell model of HD that there is increased thiol oxidization of peroxiredoxins 1, 2 and 4 implying a functionally inactive state. Further, treatment of this cell line with a dithiol compound protected against mHTT-induced toxicity and decreased the level of peroxiredoxin 1 oxidation^17^. Apoptosis signal-regulating kinase (ASK1) is a mitogen-activated protein kinase kinase kinase and an important regulator of oxidative and ER stress-induced apoptosis^58^. Inhibition of ASK1 using intra-cerebral infusion of an antibody has protective effects in mouse HD, decreasing ER stress and resulting in behavioral improvements^59^. TRX1 is a negative regulator of ASK1^60^; therefore, this is another potential mechanism of protection in our model. Therefore, while a weakness of this study is that the mechanism of protection by TRX1 in our HD mouse model is undetermined, there are several substrate proteins that are in HD-associated pathways and that could be mediating protective effects.

TMX3, in contrast to TRX1, has not previously been linked to neuroprotection for any brain disorder. However, mutations in the TMX3 gene have been linked with microphthalmia and retinal developmental anomalies^61^. TMX3 is a single domain transmembrane protein. It is primarily located in the endoplasmic reticulum (ER), with its catalytic domain in the ER lumen^62^ but is also present in the mitochondrial-associated membrane^63^. Protein substrates of TMX3 have not been reported. Huntingtin protein is associated with ER membranes and has a role in intra-cellular trafficking between the Golgi and extracellular space^64^; however, it has not been shown to be present within the ER lumen. As the TMX3 catalytic domain and N171-40Q mHTT would not be expected to be present within the same cell compartment it is improbable that the N171 fragment is a direct substrate of TMX3. However, mHTT expression does induce ER stress^65^. Increased expression of TMX3 in striatum may protect against mHTT-induced ER pathology.

In summary, we have identified TRX1 and TMX3 as proteins that decrease both mHTT levels in cultured cells and mHTT-induced striatal neuronal atrophy in HD mice. These findings support a role of thiol stress in the pathogenesis of HD. While the findings of this study are novel there are some limitations. First, lentiviral protein expression in brain was only found surrounding the needle tract with less spread than has been reported previously in rats^31^. While the findings from the morphometric analysis of neuronal cell-body size indicate protective effects, selection bias in sampling offsets the strengths of the stereologic method used. Second, while neuronal atrophy is an important feature of HD neurodegeneration^42^ this was the only outcome for which we found an effect of mHTT expression in our lentiviral model. Despite the weaknesses, the findings suggest that specific modulation of thiol homeostasis has beneficial effects in HD models. Future studies could address if increased expression of TRX1 and TMX3 globally in mouse HD brain provides protection against multiple measures of neurodegeneration.

**Primary and secondary screens for thiol-disulfide oxidoreductases that change total soluble mutant huntingtin protein levels:**
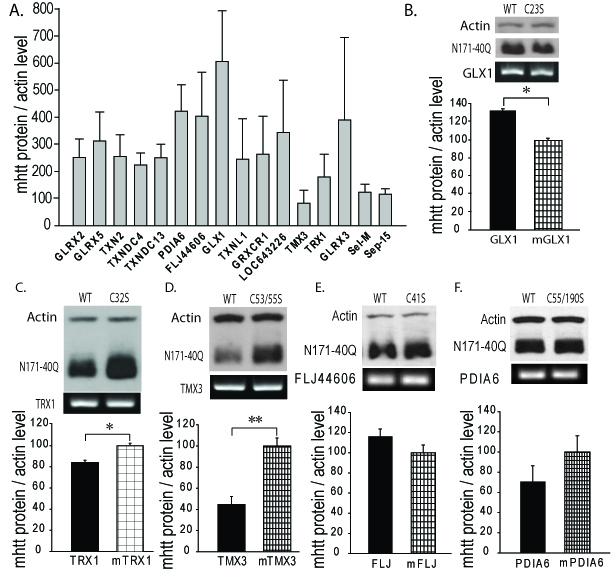
COS1 cells were transfected with plasmids encoding human thiol-disulfide oxidoreductase proteins and N171-40Q mutant huntingtin protein (mhtt). Twenty-four hours later cells were lysed and analyzed by reducing SDS-PAGE and Western blot analysis to quantify total soluble N171 mhtt. N171-40Q mutant huntingtin levels (MAB5492 – Millipore) were normalized to actin then to co-transfection control. TRX1 and TMX3 were chosen as targets that may decrease mhtt levels; glutaredoxin 1 (GLX1), PDIA6 and FLJ44606 were chosen as targets that may increase mhtt. See results for more details. Shown are mean ± SEM. n=3, B-F. Secondary screen. COS1 cells were co-transfected with plasmids encoding N171-40Q and wild-type or enzymatically non-functional (control) human thiol-disulfide oxidoreductases. Twenty-four hours later cells were lysed for reducing SDS-PAGE and western blot analysis. Candidate gene expression was confirmed by PCR. The letter/number codes above the right western blot lanes are the substitution of the mutant inactive protein. n=3-5, B. Glutaredoxin 1 (GLX1) increases soluble N171-40Q mhtt levels. n=5, C. Thioredoxin 1 (TRX1) decreases soluble N171-40Q mhtt levels. n=5, D. TMX3 decreases soluble N171-40Q mhtt levels. n=4, E. FLJ44606 has no effect on N171-40Q mhtt levels. n=4, F. Protein disulfide isomerase A6 (PDIA6) has no effect on N171-40Q mhtt levels. n=5. P-values: *<0.05 and **<0.01.

**TRX1 and TMX3 do not decrease levels of thiol-blocked N171-40Q mutant huntingtin:**
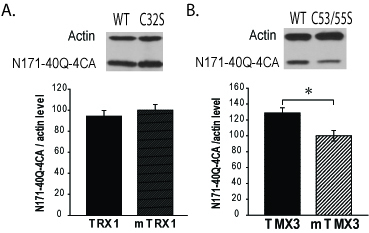
A-B. COS1 cells were co-transfected with plasmids encoding mhtt with cysteines mutated to alanines (N171-40Q-4CA) and TRX1 / TMX3. The letter/number codes above the right western blot lanes are the substitution of the mutant inactive protein. A. TRX1 does not decrease soluble N171-40Q-4CA levels. B. TMX3 increases N171-40Q-4CA levels. n=4. P-values: *<0.05.

**TRX1 does not interact directly with N171-40Q mutant huntingtin in a cell-free assay:**
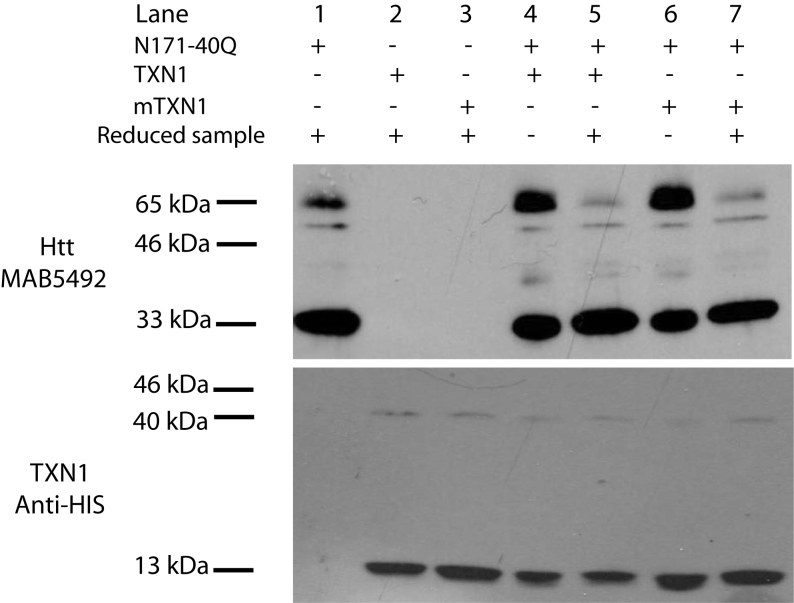
TRX1, mutant TRX1 (C35S) and N171-40Q were expressed and purified from bacteria. N171-40Q was incubated with TRX1 or mTRX1 at 250 C for 1 hour then free thiols blocked with 50 mM N-ethylmaleimide. Samples were then analyzed by SDS-PAGE (see methods). Lanes: 1=N171-40Q htt alone; 2=TRX1 alone; 3=mTRX1 alone; 4-5=N171-40Q and TRX1; and 6-7= N171-40Q and mTRX1. Mutant TRX1 (C35S) is predicted to trap substrate proteins as heterodimers [33]. There is no evidence of a heterodimer band (estimated mass = 46 kDa) in the non-reduced N171-40Q and mTRX1 group (lane 6). The band migrating at ~65 kDa (most prominent in lanes 4 and 6) is dimeric N171-40Q as previously reported^6^.

**Characterization of huntingtin expression in mouse brain following lentiviral delivery:**
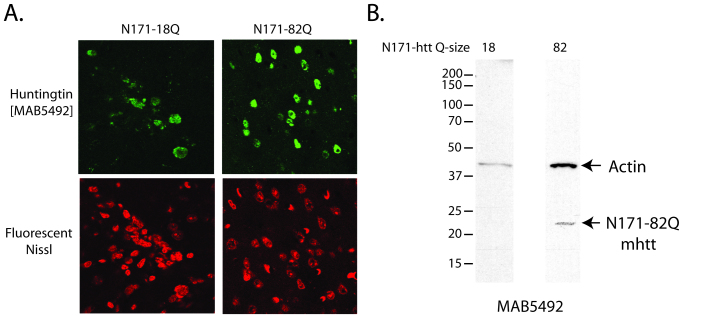
A-B. Lentivirus encoding N171 htt fragments was injected intra-striatally into 8-week-old mice; sacrifice was at 14-weeks of age. A. Detection of N171 htt in mouse striata following stereotaxic injection. Brains sections at the level of the stereotaxic injections were stained for N171 htt using MAB5492 (Chemicon) and with a fluorescent Nissl staining. Top left shows detection of 171-18Q; top right shows detection of N171-82Q; bottom show fluorescent Nissl staining. B. Detection of htt expression by Western blot analysis. Striata were dissected then homogenized to extract protein for Western blot analysis using MAB5492. N171-18Q is not detected. N171-82Q is detected.

**TMX3 expression decreases striatal neuronal atrophy in HD mice:**
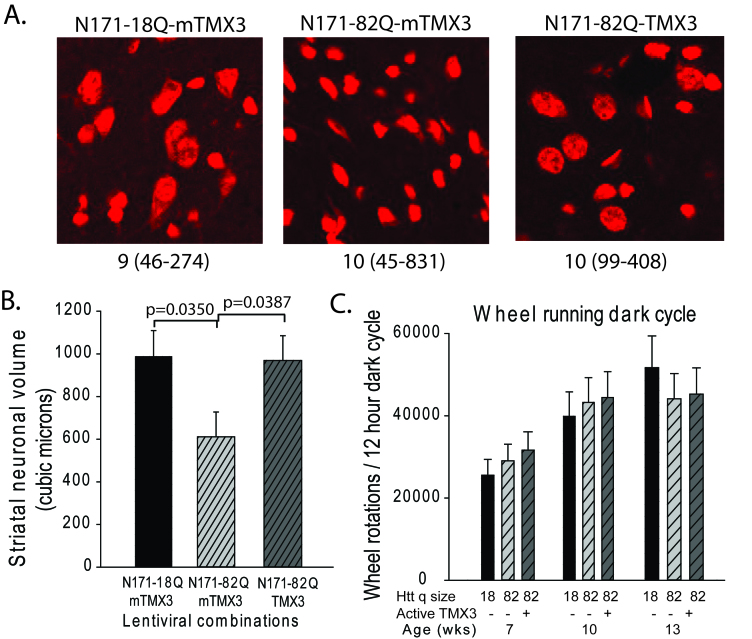
A-B. Mice were injected with lentiviral combinations (methods) at 8-weeks of age and sacrificed at 14-weeks. Brain sections were stained for Nissl substance and mhtt. Confocal images were collected in the region where htt staining was detected. Neuronal cell body volumes were determined using the nucleator method on confocal z-stack images (methods). A. Representative images of fluorescent Nissl stained neurons in the region of the striatal injection site. Numbers below images represent the number of mice per treatment group; numbers in parenthesis represent the minimum and maximum number of neurons counted per mouse within the group. B. Striatal neuronal cell body volume. Mice expressing N171-82Q and mutant (inactive) TMX3 (mTMX3) have significantly smaller neuronal cell bodies than mice expressing N171-82Q and active TMX3. The main effect p-value is 0.0346; pair-wise comparison p-values are on graph. n=9-10. C. Spontaneous wheel running activity is not altered by N171-18/82Q and / or TMX3 expression. See methods for experimental details. n=10.

**TRX1 expression decreases striatal neuronal atrophy in HD mice:**
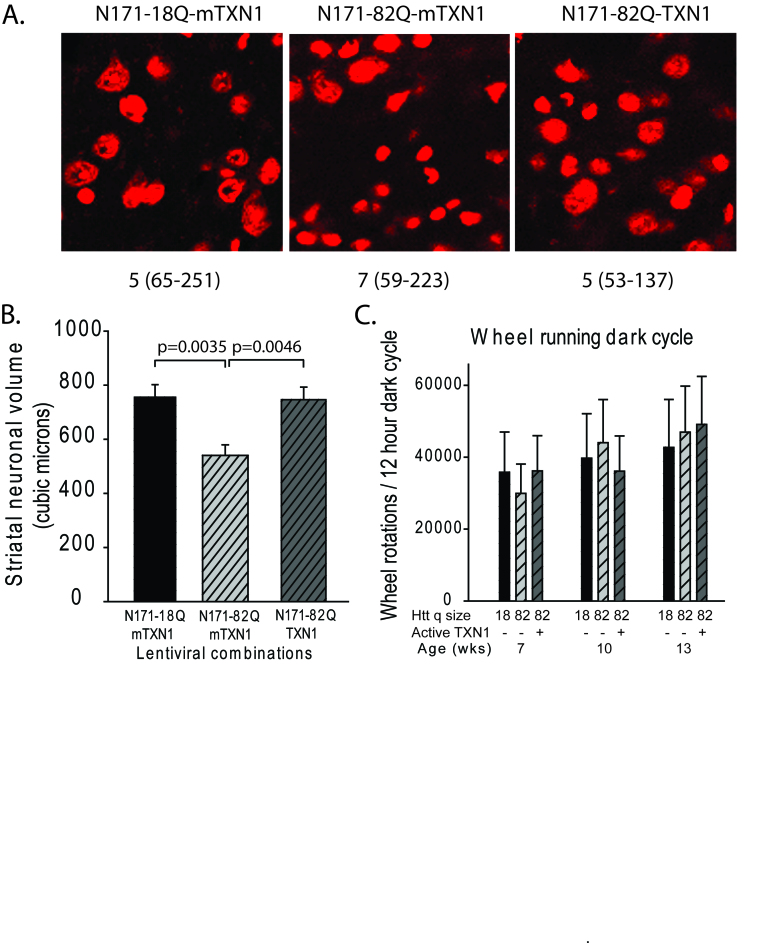
A-B. Experimental design was as described for figure 5. A. Representative images of fluorescent Nissl stained neurons in the region of the striatal injection site. Numbers below images represent the number of mice per treatment group; numbers in parenthesis represent the minimum and maximum number of neurons counted per mouse within the group. B. Striatal neuronal cell body volume. Mice expressing N171-82Q and mutant (inactive) TRX1 (mTRX1) have significantly smaller neuronal cell bodies than mice expressing N171-82Q and active TRX1. The main effect p-value is 0.0545; pair-wise comparison p-values are on graph. n=5-7, C. Spontaneous wheel running activity is not altered by N171-18/82Q and / or TXN1 expression. See methods for experimental details. n=7-9.

**TRX1 and TMX3 transcript levels in transgenic N171-82Q HD mouse brain:**
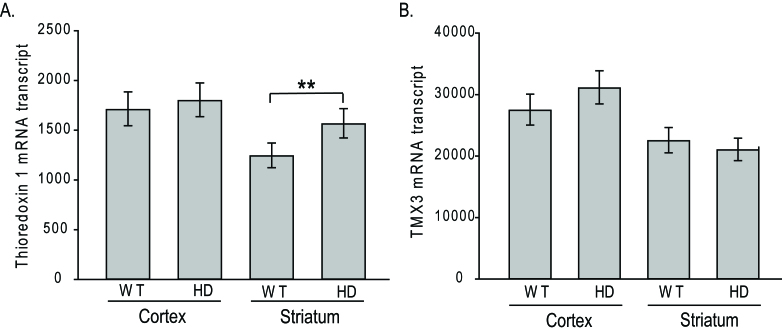
A. Transcript-encoding TRX1 in HD mice is significant higher than WT littermates in striatum, but not cortex. B. Transcript-encoding TMX3 in HD mice is not altered compared to WT littermates in striatum and cortex. All analyses were in 14-week-old mice corresponding to early-advanced disease. Values are normalized to actin. Shown are means ± 95% confidence interval. n=9-10, p=value: **<0.01.

## List of abbreviations

Mutant huntingtin protein, mhtt; Huntington’s disease, HD; endoplasmic reticulum, ER; sodium dodecyl sulfate, SDS; thioredoxin-related transmembrane protein 3, TMX3; thioredoxin 1, TRX1; protein disulfide isomerase family A, member 6, PDIA6.

## Competing interest statement

I have read the journal’s policy and have the following conflict. Application serial number 13/854,809 filed with US patent office.

## Author contributions

ZL carried out cell culture studies, synthesized plasmid constructs, and assisted with the mouse experiments and writing of the paper. LB carried out the viral synthesis and mouse experiments. JF conceived, designed and coordinated the study and wrote the paper. All authors read and approved the final manuscript.
