# Correction: Thiol-disulfide Oxidoreductases TRX1 and TMX3 Decrease Neuronal Atrophy in a Lentiviral Mouse Model of Huntington’s Disease

**DOI:** 10.1371/currents.hd.30bdfed3d88457fd605cb8f95fba29b5

**Published:** 2016-01-07

**Authors:** - PLOS Currents

## Correction

The author order is incorrect. The correct order of the authors is: Lu Z, Barrows L, Fox J.

## Reference

Fox J, Lu Z, Barrows L. Thiol-disulfide Oxidoreductases TRX1 and TMX3 Decrease Neuronal Atrophy in a Lentiviral Mouse Model of Huntington’s Disease. PLOS Currents Huntington Disease. 2015 Nov 6 . Edition 1. doi: 10.1371/currents.hd.b966ec2eca8e2d89d2bb4d020be4351e.

